# Supplementary material for: Evaluation of phytochemical profile, and antioxidant, antidiabetic activities of indigenous Thai fruits
Source: PeerJ. 2024 Jul 12;12:e17681. doi: 10.7717/peerj.17681 (PMC11249001; doi:10.7717/peerj.17681)
Supplement: Supplemental Information 1 — Gallic acid (1), Catechin (2), Epicatechin (3), Epicatechin gallate (4), Ellagic acid (5), and Kaempferol (6). [file peerj-12-17681-s001.docx]

**UHPLC-DAD chromatograms of fruit extracts**, Gallic acid (1), Catechin (2), Epicatechin (3), Epicatechin gallate (4), Ellagic acid (5), and Kaempferol (6).

| 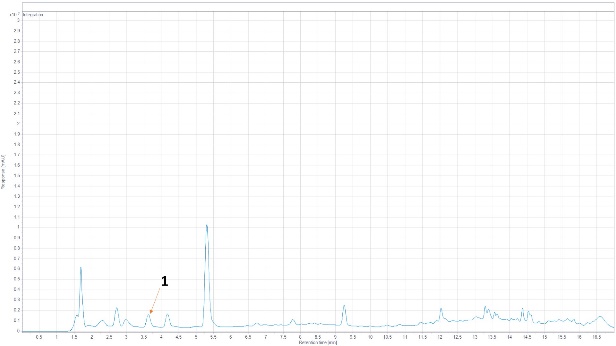 | 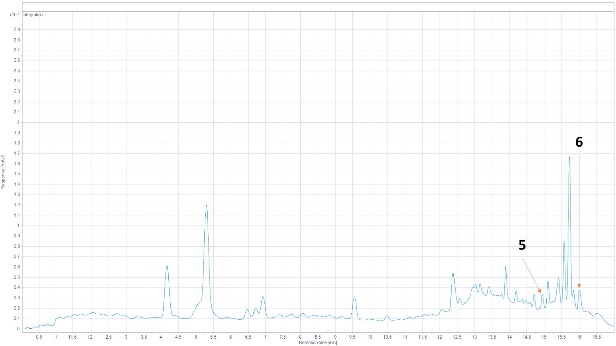 |
| --- | --- |
| *A. puncticulatum* | *D. indica* |
| 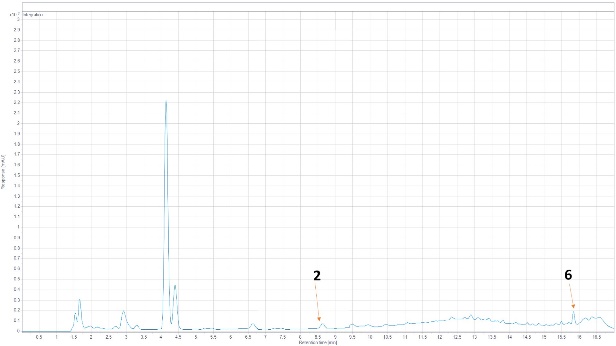 | 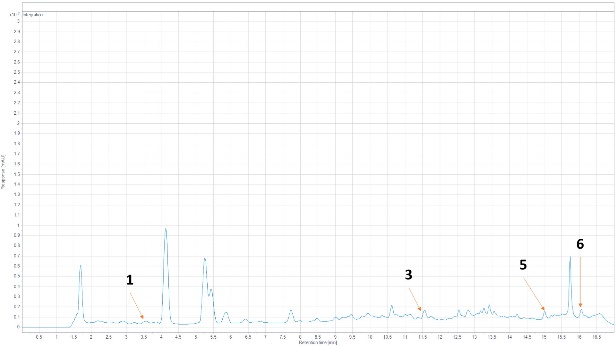 |
| *D. decandra* | *E. latifolia* |

| 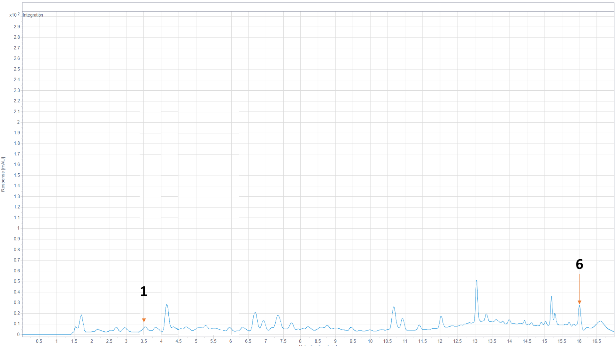 | 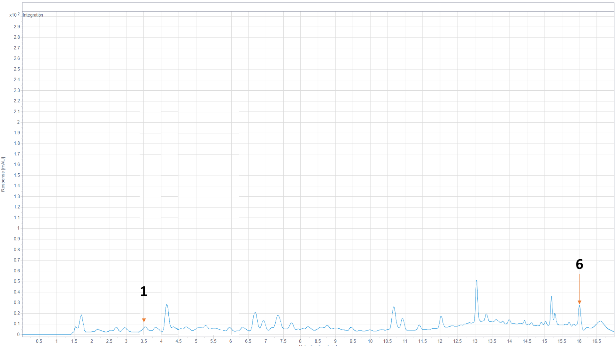 |
| --- | --- |
| *F. indica* | *G. dulcis* |
| 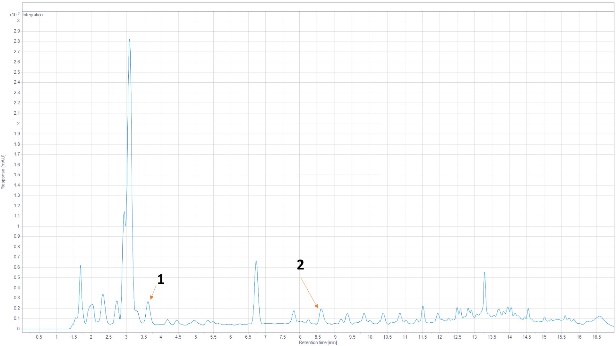 | 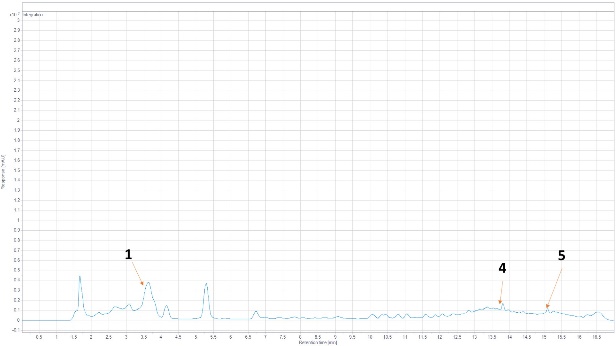 |
| *L. fruticose* | *M.s elengi* |

| 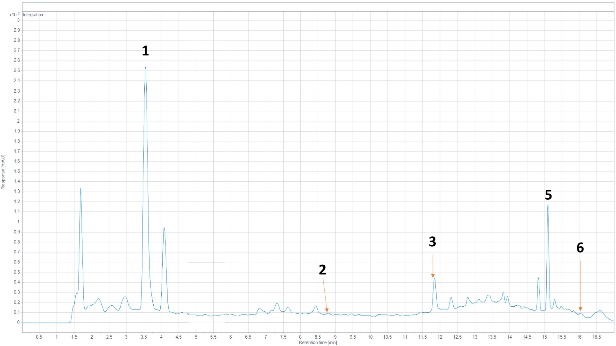 | 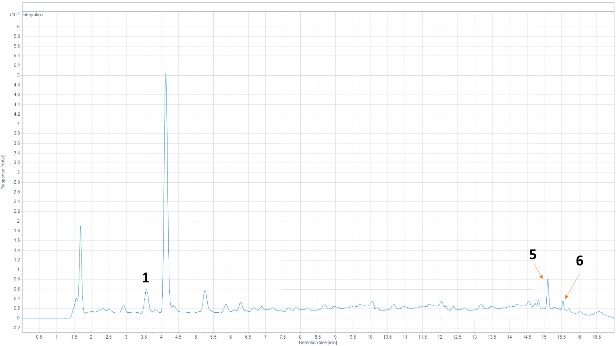 |
| --- | --- |
| *M. calabura* | *P. reticulatus* |
| 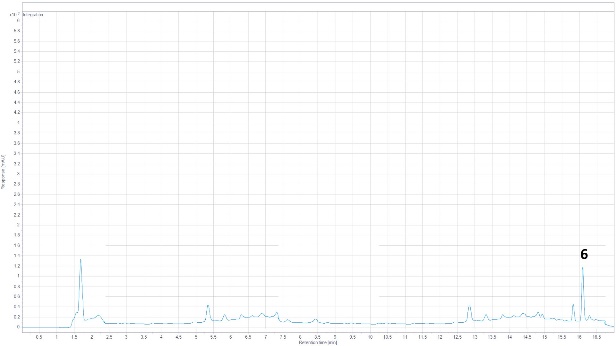 | 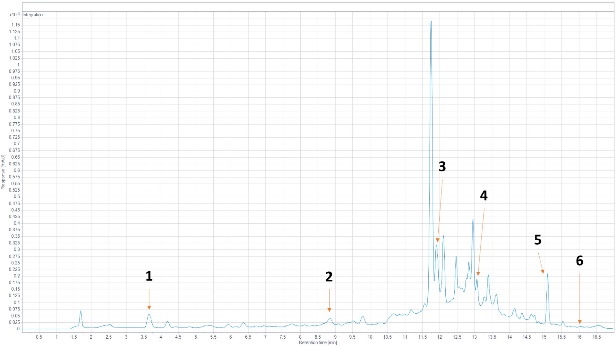 |
| *S. asper* | *S. cumini* |

| 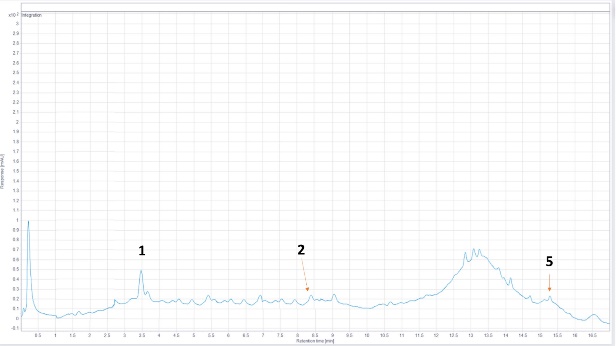 | 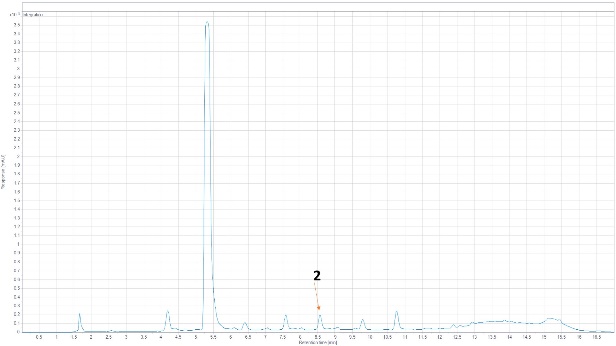 |
| --- | --- |
| *S. malaccense* | *S. malaccense* |
| 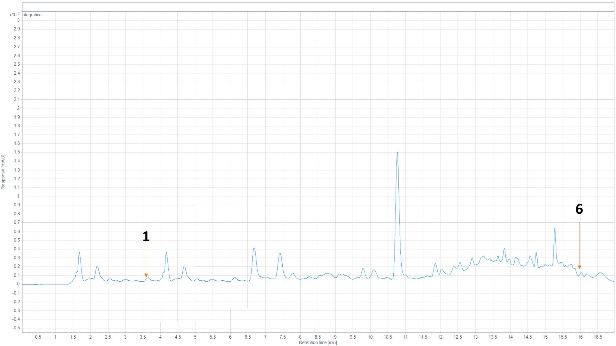 |  |
| *W. edulis* |  |
